# Genetic dissection of susceptibility genes for diabetes and related phenotypes on mouse chromosome 14 by means of congenic strains

**DOI:** 10.1186/s12863-014-0093-8

**Published:** 2014-08-29

**Authors:** Naru Babaya, Hironori Ueda, Shinsuke Noso, Yoshihisa Hiromine, Michiko Itoi-Babaya, Misato Kobayashi, Tomomi Fujisawa, Hiroshi Ikegami

**Affiliations:** 1 Department of Endocrinology, Metabolism and Diabetes, Kinki University School of Medicine, 377-2 Ohno-higashi, Osaka-sayama 589-8511, Osaka, Japan; 2 Department of Molecular Endocrinology, Osaka University Graduate School of Medicine, Suita 565-0871, Osaka, Japan; 3 Department of Geriatric Medicine and Nephrology, Osaka University Graduate School of Medicine, Suita 565-0871, Osaka, Japan; 4 Department of Applied Molecular Bioscience, Graduate School of Bioagricultural Sciences, Nagoya University, Nagoya 464-8601, Aichi, Japan

**Keywords:** Adiposity, Animal model, Complex trait, Consomic strain, Insulin resistance

## Abstract

**Background:**

A susceptibility locus, *Nidd2n*, for type 2 diabetes has been mapped to mouse chromosome 14 (Chr 14) and confirmed using the consomic strain (C3H-Chr 14^NSY^) of the Nagoya-Shibata-Yasuda (NSY) mouse, an animal model of spontaneous type 2 diabetes. The aim of this study was to localize and characterize *Nidd2n*.

**Results:**

We constructed two novel congenic strains homozygous for different segments of NSY-Chr 14 on the control C3H/HeNcrj (C3H) background: R1 (C3H.NSY-(*D14Mit206-D14Mit5*)) possesses the proximal and middle segment, and R2 (C3H.NSY-(*D14Mit206-D14Mit186*)) possesses the most proximal segment of NSY-Chr 14. Diabetes-related phenotypes were studied in comparison with those of consomic C3H-Chr 14^NSY^ (R0) and parental NSY and C3H strains. Congenic R1 and R2 showed significantly higher post-challenge glucose than that in C3H mice. Fasting glucose, in contrast, was significantly lower in R1 and R2 than in C3H mice. Insulin sensitivity was significantly impaired in R1 and R2 compared to C3H mice. R2 showed significantly higher body weight and fat-pad weight than those in C3H and R1. Leptin level was significantly higher in R0, R1 and R2 than in C3H mice, with R2 showing the highest level, similar to that in NSY mice. Serum adiponectin level was significantly lower in R0, R1 and R2 than in C3H mice, while it was significantly higher in NSY than in C3H mice.

**Conclusions:**

These data indicate that Chr 14 harbors multiple genes for diabetes-related phenotypes. The original *Nidd2n*, which is located in the middle region of Chr 14, was divided into two segments; *Nidd2.1n* in proximal Chr 14 and *Nidd2.2n* in distal Chr 14. *Nidd2.1n* contributes to post-challenge hyperglycemia, insulin resistance and adiposity. *Nidd2.2n* contributes to fasting as well as post-challenge hyperglycemia and insulin resistance. *Adp1n*, which contributes to decreased adiposity and increased insulin sensitivity, rather than a diabetogenic gene, was mapped in the middle segment.

## Background

Diabetes and obesity are multifactorial diseases caused by a complex interaction of environmental and genetic factors, with the latter consisting of multiple susceptibility genes, making it difficult to clarify their functions and interactions in conferring susceptibility to diabetes and obesity in humans. Recent genome-wide association studies (GWAS) have identified a large number of single nucleotide polymorphisms associated with diabetes and obesity [1], but identification of the causal variants in these loci is a formidable challenge. Inbred animal models of diabetes and obesity are therefore invaluable to decipher the complexity of human diabetes and obesity, as evidenced by the identification of common functional variants of a gene involved in type 1 diabetes susceptibility in both humans and mice [2].

The Nagoya-Shibata-Yasuda (NSY) mouse was established as an inbred animal model with spontaneous development of type 2 diabetes by selective breeding for glucose intolerance from a closed colony of Jcl:ICR mice [3]-[5]. The NSY mouse shares many features of diabetes with human type 2 diabetes in that the onset is age-dependent, the disease is associated with moderate obesity with abdominal fat accumulation, and both impaired insulin response to glucose and insulin resistance contribute to the disease development [4],[5].

Using an F2 cross of the NSY and control C3H/HeNcrj (C3H) strains, we previously mapped three major quantitative trait loci (QTLs) affecting diabetes-related phenotypes (*Nidd1n*, *Nidd2n* and *Nidd3n* on Chr 11, 14 and 6, respectively) [6], a QTL for fatty liver (*Fl1n* on Chr 6) and a QTL for body weight (*Bw1n* on Chr 7) [7]. To obtain direct evidence that *Nidd2n* on Chr 14 confers susceptibility to diabetes, we constructed consomic C3H-Chr 14^NSY^ mice, in which the NSY-derived whole Chr 14 was introgressed onto the genetic background of control C3H mice [8], because the regions showing significant linkage with *Nidd2n* were broad (peak region near *D14Mit5*) [6]. C3H-Chr 14^NSY^ mice showed significantly higher blood glucose levels than those in C3H mice, indicating that Chr 14 harbors a locus for hyperglycemia. C3H-Chr 14^NSY^ mice showed significantly impaired insulin sensitivity, but normal insulin secretion, indicating that the main effect was due to insulin resistance [8]. Body weight was not increased by introgression of NSY-Chr 14 alone, but did increase in the presence of NSY-Chr11, indicating a genetic interaction between Chr 14 and Chr 11 for obesity [8]. The region on mouse Chr 14 is syntenic to human Chr 3p, 8p, 10q, 13q, and 14q, and several whole genome studies have mapped loci associated with diabetes [9]-[12] and obesity [13] to the corresponding regions. Although *Nidd2n* has a broad peak in the odds curve, the nearest marker of the peak region is *D14Mit5*[6] on the C3 region of the chromosomal band, which is syntenic to human 13q12. Many genome-wide association studies have revealed several candidate genes for type 2 diabetes [1], although the orthologues of these genes are not located near *Nidd2n* or on Chr 14. Identification and characterization of the function of *Nidd2n* on mouse Chr 14 are expected to contribute to identification and characterization of diabetogenic genes in humans.

The present study was performed to localize and characterize the function of *Nidd2n* on mouse Chr 14. To this end, we constructed two novel congenic strains homozygous for different segments of NSY-Chr 14 and investigated diabetes-related phenotypes in these mice in comparison with those of the original consomic C3H-Chr 14^NSY^ and parental NSY and C3H strains.

## Methods

### Animals

The consomic strain, C3H-Chr 14^NSY^ (R0), homozygous for NSY-derived whole Chr 14 on the control C3H/HeNcrj (C3H) background, was previously established in the N8F1 generation using the speed congenic method [8]. Congenic lines were produced by mating (R0 x C3H) F1 with C3H and selecting males that possessed the genomic region of interest on Chr 14 using 9 microsatellite markers (Additional file 1: Table S1). For the background genome, we used 69 microsatellite markers (Additional file 1: Table S1) spanning the whole mouse genome except for Chr 14, and confirmed the markers derived from C3H, as described previously [8],[14]. These male mice were mated with female C3H mice, and their progeny with the genomic region of interest were intercrossed to obtain homozygotes. These lines were maintained by brother-sister mating.

Mice were maintained under specific pathogen-free (SPF) conditions in the animal facilities of Osaka University Graduate School of Medicine. All mice had free access to tap water and a standard diet (CRF-1: Oriental Yeast, Tokyo, Japan) in an air-conditioned room (22–25°C) with a 12-h light–dark cycle (6:00–18:00 h). Mice were housed in PC7115HT cages, 189 mm × 297 mm × 128 mm (Allentown Inc., New Jersey, USA), at 6 or fewer mice per cage. The animal protocols used for this study were approved by the Osaka University Graduate School of Medicine Committee on Animal Welfare (Approval number: 030038–444).

### Phenotypic analyses

Glucose tolerance and body weight were monitored at 24 weeks of age. Glucose tolerance was assessed by intraperitoneal glucose tolerance test (ipGTT) (2 g glucose/kg body weight) in overnight-fasted mice, and blood glucose levels were measured at 0, 30, 60, 90, and 120 min. The area under the glucose concentration curve (gAUC) was calculated according to the trapezoid rule from the glucose measurements at baseline (0 min), 30, 60, 90, and 120 min.

Insulin tolerance test (ITT) was performed by injecting human insulin (0.25 IU/kg body weight) intraperitoneally in overnight-fasted mice at 26 weeks of age, and blood glucose levels were measured at 0, 15, 30, 45, and 60 min. Results are expressed as the % decrease in glucose area from the baseline.

Insulin secretion in response to glucose was assessed by ipGTT (2 g glucose/kg body weight) in overnight-fasted mice at 28 weeks of age, and blood glucose and serum insulin levels were measured at 0, 15, and 30 min. Incremental AUC of insulin (ΣΔiAUC) and glucose (ΣΔgAUC) were calculated according to the trapezoid rule from the insulin and glucose measurements at baseline (0 min), 15, and 30 min. The insulinogenic index was calculated as ΣΔiAUC ÷ ΣΔgAUC.

Anatomical phenotypes were studied at 30 weeks of age. Under anaesthesia with pentobarbital (Dainippon, Osaka, Japan), body weight and anal-nasal length were measured. BMI was calculated as body weight in g divided by the square of anal–nasal length in cm. Mice were killed under sevoflurane anaesthesia, and the epididymal, mesenteric and retroperitoneal fat pads were dissected out and weighed.

### Blood sample assays

Blood glucose level was determined by the glucose oxidase method using Glutest Ace (Sanwa Kagaku Kenkyusho Co., Ltd., Nagoya, Japan). Plasma insulin level was measured by ELISA (Morinaga, Yokohama, Japan). Insulin values in micrograms per liter obtained by ELISA were converted to picomoles per liter by multiplying by 174. Serum leptin and adiponectin were measured at 30 weeks of age with an ELISA-based leptin assay (TECHNE, Minneapolis, USA) and adiponectin assay (Otsuka Pharmaceutical Co., Ltd., Tokyo, Japan), according to the manufacturers’ instructions.

### Statistical analysis

All results are expressed as mean ± SEM. Statistical analysis was performed by unpaired *t*-test. Correlations between leptin and fat-pad weight and between adiponectin and fat-pad weight were examined with the Pearson correlation coefficient. A value of p <0.05 was regarded as significant.

## Results

### Establishment of new congenic strains

**Figure 1:**
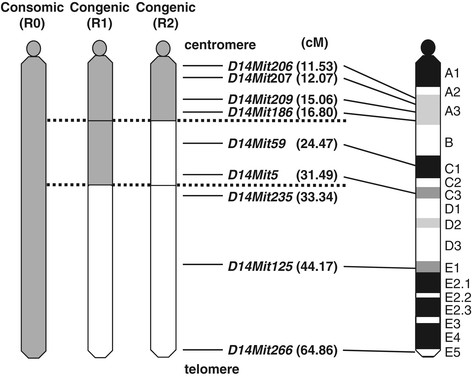
**Schematic illustration of chromosome 14 of consomic mice (R0 (C3H-Chr 14**^**NSY**^**)) and congenic mice (R1 (C3H.NSY-(*D14Mit206-D14Mit5*)), R2 (C3H.NSY-(*D14Mit206-D14Mit186*))), which carry NSY-derived susceptible regions on a C3H-derived resistance background.** Regions from NSY mice are shown in gray, and regions from C3H mice are shown in white. The map positions of simple sequence length polymorphisms from the centromere shown in parentheses (cM) were obtained from the Mouse Genome Database (www.informatics.jax.org).

We produced two novel congenic strains, which possessed different segments of NSY-derived Chr 14 (Figure 1). The congenic strain R1 (C3H.NSY-(*D14Mit206-D14Mit5*)) possessed the proximal and middle segment of NSY-Chr 14 from the centromere to the recombinant position between *D14Mit5* and *D14Mit235*. The other congenic strain R2 (C3H.NSY-(*D14Mit206-D14Mit186*)) possessed the proximal segment of NSY-Chr 14 from the centromere to the recombinant position between *D14Mit186* and *D14Mit59*.

### Phenotypic analysis of consomic C3H-Chr 14^NSY^; R0

**Figure 2:**
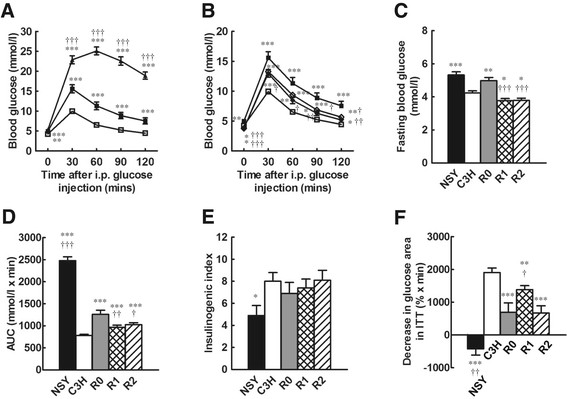
**Diabetes-related phenotypes in NSY, C3H, C3H-Chr 14**^**NSY**^**(R0), C3H.NSY-(*D14Mit206-D14Mit5*) (R1) and C3H.NSY-(*D14Mit206-D14Mit186*) (R2) mice. A**. Blood glucose levels during ipGTT in NSY (*n =* 16; ▲), C3H (*n =* 39; □) and R0 mice (*n =* 36; ■). **B**. Blood glucose levels during ipGTT in C3H (*n =* 39; □), R0 (*n =* 36; ■), R1 (*n =* 23; ◆) and R2 mice (*n =* 23; ◇). **C**. Fasting glucose level in NSY (*n =* 16), C3H (*n =* 39), R0 (*n =* 36), R1 (*n =* 23) and R2 mice (*n =* 23). **D**. Area under glucose concentration curve (gAUC) during ipGTT in NSY (*n =* 16), C3H (*n =* 39), R0 (*n =* 36), R1 (*n =* 23) and R2 mice (*n =* 23). **E**. Insulinogenic index in NSY (*n =* 20), C3H (*n =* 21), R0 (*n =* 15), R1 (*n =* 20) and R2 mice (*n =* 16). **F**. Insulin resistance assessed by decrease in glucose area in insulin tolerance test in NSY (*n =* 18), C3H (*n =* 18), R0 (*n =* 19), R1 (*n =* 23) and R2 mice (*n =* 22). ^*^p < 0.05, ^**^p < 0.01, ^***^p < 0.001 vs. C3H. ^†^*p <* 0.05, ^††^*p <* 0.01, ^†††^*p <* 0.001 vs. R0.

R0 mice exhibited significantly higher blood glucose levels after fasting (p < 0.01) and at all time points after a glucose challenge (p < 0.001) than those in C3H mice (Figure 2A). The insulinogenic index in R0 mice was similar to that in C3H mice, and was significantly higher than that in NSY mice (p < 0.05) (Figure 2E). The glucose-lowering effect of insulin was significantly impaired in R0 compared with that in C3H mice (p < 0.001) (Figure 2F). No significant difference was observed in body weight and BMI between R0 and C3H mice (Table 1). Fat-pad weight and the percentage of fat-pad weight/body weight were significantly elevated in R0 compared with those in C3H mice (p < 0.01).

**Table 1:** Anatomical analysis of parental NSY and C3H mice, consomic R0 and congenic R1 and R2 mice at 30 weeks of age

	**Parental strains**		**Consomic strain**	**Congenic strains**	
	**NSY**	**C3H**	**R0**	**R1**	**R2**
Number of mice analyzed	9	20	15	12	15
Body weight (g)	43.5 ± 1.6^c^	29.5 ± 0.5	30.2 ± 0.6	30.8 ± 0.8	32.2 ± 0.6^c,d^
Anal-nasal length (cm)	11.0 ± 0.1^c^	10.2 ± 0.1	10.1 ± 0.1	10.1 ± 0.1	10.2 ± 0.1
BMI (g/cm^2^)	0.357 ± 0.007^c^	0.283 ± 0.005	0.294 ± 0.005	0.304 ± 0.005^b^	0.310 ± 0.005^c,d^
Total fat (g)	3.451 ± 0.260^c^	1.023 ± 0.052	1.385 ± 0.100^b^	1.390 ± 0.088^c^	1.683 ± 0.095^c,d,f^
Epididymal fat (g)	1.578 ± 0.106^c^	0.509 ± 0.033	0.723 ± 0.052^b^	0.730 ± 0.058^b^	0.880 ± 0.051^c,e^
Retroperitoneal fat (g)	1.083 ± 0.120^c^	0.119 ± 0.009	0.179 ± 0.018^b^	0.173 ± 0.013^b^	0.269 ± 0.019^c,e,g^
Mesenteric fat (g)	0.789 ± 0.059^c^	0.395 ± 0.020	0.483 ± 0.033^a^	0.487 ± 0.025^b^	0.534 ± 0.029^c^
Total fat / body weight (%)	7.85 ± 0.34^c^	3.49 ± 0.18	4.53 ± 0.26^b^	4.48 ± 0.22^b^	5.18 ± 0.21^c,f^

Values are total number or mean ± SEM.

NSY, C3H-Chr 14^NSY^ (R0), C3H.NSY-(*D14Mit206-D14Mit5*) (R1), and C3H.NSY-(*D14Mit206-D14Mit186*) (R2) mice were compared with C3H (^a^*p <* 0.05, ^b^*p <* 0.01, ^c^*p <* 0.001). R1 and R2 mice were compared with R0 (^d^*p <* 0.05, ^e^*p <* 0.01). R2 was compared with R1 (^f^*p <* 0.05, ^g^*p <* 0.001).

### Phenotypic analysis of congenic R1 mice

Fasting glucose in R1 mice was slightly, but significantly lower than that in control C3H mice (p < 0.05) (Figure 2B, C). In contrast, glucose levels at all time points after a glucose challenge were significantly higher in R1 mice than in C3H mice (Figure 2B). Hyperglycemia in R1 mice, as assessed by AUC during ipGTT, was significantly more severe than that in C3H mice, but was not as severe as that in R0 mice (p < 0.01) (Figure 2D). Insulinogenic index was similar in C3H, R0 and R1 mice, with no significant difference (Figure 2E). In contrast, the glucose-lowering effect of insulin was significantly impaired in R1 compared to C3H mice (p < 0.01), but this was not as severe as in R0 mice (p < 0.05) (Figure 2F).

Body weight in R1 mice was not significantly different from that in C3H mice, as in the case of R0 mice. Fat-pad weight and percentage fat-pad weight/body weight were significantly higher in R1 mice than in C3H mice (Table 1). No significant differences were observed between R1 and R0 mice (Table 1).

### Phenotypic analysis of congenic R2 mice

Glucose tolerance in R2 mice was similar to that in R1 mice (Figure 2B, C, D). Insulinogenic index in R2 mice was not significantly different from that in C3H mice (Figure 2E). The glucose-lowering effect of insulin during ITT was significantly impaired in R2 compared with that in C3H mice (p < 0.001) and R1 mice (p < 0.01) (Figure 2F).

R2 mice showed significantly higher body weight, BMI, fat-pad weight and percentage fat-pad weight/body weight than those in control C3H mice (Table 1). Body weight, BMI and fat-pad weight were significantly higher in R2 mice than in R0 mice (Table 1). Fat-pad weight and percentage fat-pad weight/body weight in R2 mice were significantly higher than those in R1 mice (Table 1).

### Adipocytokines

**Figure 3:**
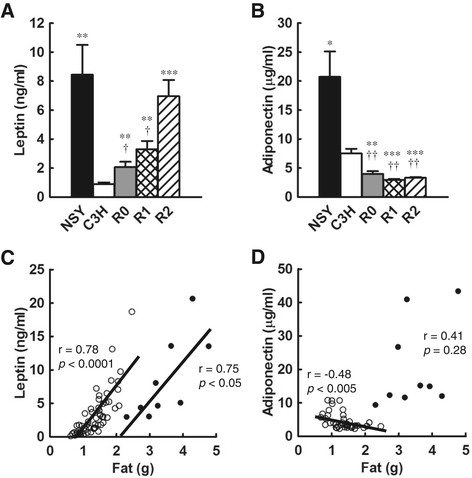
**Adipocytokines in NSY, C3H, C3H-Chr 14**^**NSY**^**(R0), C3H.NSY-(*D14Mit206-D14Mit5*) (R1) and C3H.NSY-(*D14Mit206-D14Mit186*) (R2) mice. A**. Serum leptin level in NSY (n = 9), C3H (n = 17), R0 (n = 15), R1 (n = 12) and R2 mice (n = 15). **B**. Serum adiponectin level in NSY (n = 9), C3H (n = 11), R0 (n = 11), R1 (n = 11) and R2 mice (n = 11). **C**. Correlation between leptin and visceral fat-pad weight in mice with C3H background (C3H, R0, R1, and R2 mice; n = 59; ○) and NSY mice (n = 9; ●). Lines indicate linear regression. **D**. Correlation between adiponectin level and visceral fat-pad weight in mice with C3H background (C3H, R0, R1, and R2 mice; n = 44; open circles) and NSY mice (n = 9; closed circles). Lines indicate linear regression. ^*^p < 0.05, ^**^p < 0.01, ^***^p < 0.001 vs. C3H. ^†^*p <* 0.05, ^††^*p <* 0.01, ^†††^*p <* 0.001 vs. NSY.

Serum leptin level was significantly higher in NSY than in C3H mice. R0, R1 and R2 mice showed significantly higher leptin level than that in C3H mice (Figure 3A). Leptin level in R0 and R1 mice was significantly lower than that in NSY mice, while that in R2 mice was not significantly different from that in NSY mice (Figure 3A).

Serum adiponectin level in NSY mice was significantly higher than that in C3H mice (Figure 3B), despite their having a much higher body mass index and fat mass (Table 1), which usually reduce serum adiponectin level. Adiponectin level in R0, R1 and R2 mice was significantly lower than that in C3H mice, with no significant difference among R0, R1 and R2 mice (Figure 3B).

Since serum levels of leptin and adiponectin are known to be affected by fat mass, we studied the correlation of serum leptin and adiponectin levels with fat-pad weight (Figure 3C, D). A significant correlation was observed between leptin level and fat-pad weight in mice with C3H background (R0, R1, R2 and C3H mice) (r = 0.78, *p <* 0.0001). A significant correlation with a similar slope was also observed in NSY mice (r = 0.75, *p <* 0.05), but the regression line was shifted to the right (Figure 3C).

A significant negative correlation between adiponectin level and fat-pad weight was observed in mice with C3H background (R0, R1, R2 and C3H mice; r = −0.48, *p <* 0.005), but not in NSY mice (r = 0.41, *p =* 0.28) (Figure 3D).

## Discussion

**Figure 4:**
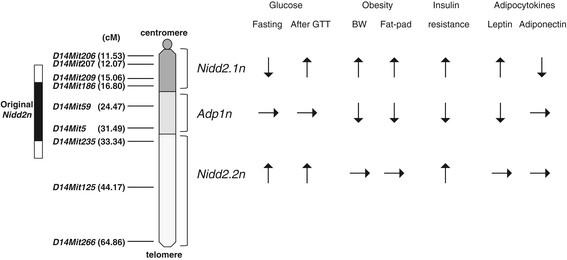
**Position of diabetes-related loci,** ***Nidd2.1n*,** ***Nidd2.2n*** **and** ***Adp1n*, on mouse chromosome 14 and their effect on diabetes-related phenotypes.** Original *Nidd2n* is shown in black (significant linkage) and white (suggestive linkage) boxes. ↑; increase, ↓; decrease.

We previously mapped a QTL, *Nidd2n*, affecting diabetes-related phenotypes in a broad segment of Chr 14 [6]. Subsequent studies using consomic C3H-Chr 14^NSY^ mice, in which the entire NSY-Chr 14 was introgressed onto the genetic background of control C3H mice, clearly demonstrated that NSY-Chr 14 harbors a locus for diabetes and insulin resistance without body weight gain [8]. To further localize and characterize *Nidd2n* on Chr 14, we established two novel congenic strains, in which limited segments of NSY-Chr 14 were introgressed onto control C3H background genes. One congenic strain, termed R1, possessed the proximal half segment of NSY-Chr 14, and the other congenic strain, termed R2, possessed a more limited segment of NSY-Chr 14 (Figure 1). By comparing the phenotypes of congenic R1 and R2 mice with those of original C3H-Chr 14^NSY^ consomic mice, termed R0, as well as those of parental NSY and C3H mice, we found that *Nidd2n* consisted of at least two components, one in the proximal region and the other in the distal region of Chr 14. Since R2 mice, which possessed the most proximal segment of NSY-Chr 14, showed significantly higher blood glucose levels after glucose challenge than those in control C3H mice, one component of *Nidd2n* affecting hyperglycemia is localized to this region. The fact that R1 mice, which possessed a larger segment of proximal NSY-Chr 14, showed similar glucose levels to those in R2 mice, but significantly lower glucose levels than those in consomic R0 mice, suggests that a second component of *Nidd2n* affecting hyperglycemia is localized to the distal segment of NSY-Chr 14, which is possessed by R0, but not by R1. We provisionally designated the former component *Nidd2.1n* and the latter component *Nidd2.2n* (Figure 4).

In contrast to the consistently high post-challenge glucose levels in all strains with NSY-Chr 14, namely R0, R1 and R2, fasting glucose level in R1 and R2 mice was completely different from that in R0 mice in that R1 and R2 mice showed a significantly lower fasting glucose level than that in C3H mice, while R0 mice showed a significantly higher level than that in C3H mice, as in the case of NSY mice. These data indicate that the genetic control of fasting glucose and post-challenge glucose is different, and that the distal segment of NSY-Chr 14 possessed by R0, but not by R1 and R2, harbors a locus for fasting hyperglycemia. As discussed in the previous section, this segment harbors *Nidd2.2n*, which increases post-challenge glucose from the levels in R1 and R2 mice to the higher levels observed in R0 mice. Thus, *Nidd2.2n* in the distal segment of NSY-Chr 14 affects fasting as well as post-challenge hyperglycemia, while *Nidd2.1n* in the proximal segment of NSY-Chr 14 affects only post-challenge hyperglycemia, but not fasting hyperglycemia, and rather decreases the fasting glucose level.

Consistent with our previous observation in R0 consomic mice [8], insulin secretion in response to glucose loading was not impaired in either the R1 or R2 congenic strain. In contrast, both R1 and R2 mice were insulin resistant as shown by the decrease in the glucose-lowering effect of insulin, as in the case of consomic R0. These data suggest that hyperglycemia in R0, R1 and R2 mice is mainly caused by insulin resistance, rather than impaired insulin secretion, and that a locus for insulin resistance is localized in the proximal segment of Chr 14 retained in R2 mice. This region overlaps with the *Nidd2.1n* region affecting hyperglycemia described above (Figure 4), suggesting that *Nidd2.1n* is a locus for insulin resistance as well as hyperglycemia.

Anatomical analysis revealed that phenotypes of R2 congenic mice are notably different from those of R0 and R1 mice. In contrast to the almost similar body weight and adiposity in R0 and R1 mice, R2 mice showed significantly greater body weight than R0 mice and greater fat-pad weight than R0 and R1 mice (Table 1). These data indicate that a locus that strongly increases adiposity is located in the proximal segment of NSY-Chr 14 retained in R2 mice, and that a locus outside this region of Chr 14 plays a role in the reduction of body weight and adiposity. The former locus may well be the same as *Nidd2.1n* for insulin resistance and hyperglycemia discussed above. The latter locus, which is protective against obesity and adiposity, is localized to the middle segment of NSY-Chr 14 possessed by R1, but not by R2, and is provisionally designated *Adp1n* for *ad*i*p*osity locus *1* in *N*SY (Figure 4). The NSY allele at *Adp1n* decreases while the C3H allele increases fat-pad weight. *Adp1n* may explain why R0 consomic mice in our previous study were insulin resistant without body weight gain, in that insulin resistance and adiposity were controlled by *Nidd2.1n*, while adiposity was reduced and obesity was masked by the effect of *Adp1n*. Mice with NSY alleles at both *Nidd2.1n* and *Adp1n*, as in the case of R0 and R1, show insulin resistance without body weight gain, while mice with the NSY allele at *Nidd2.1n*, but with the C3H allele at *Adp1n*, as in the case of R2, show insulin resistance with obesity.

Although all three strains, R0, R1 and R2, showed insulin resistance as compared with control C3H mice, insulin resistance was significantly milder in R1 than in R0 mice (Figure 2F), suggesting that a locus in the distal segment of NSY-Chr 14 possessed by R0, but not by R1, contributed to increased insulin resistance. These data suggest that at least three loci on Chr 14 contribute to insulin resistance: one in the proximal, another in the middle and the other in the distal segment (Figure 4). An NSY allele at a locus in the middle segment of Chr 14 contributed to the reduction of insulin resistance observed in R1, while NSY alleles in the proximal and distal segments contributed to worse insulin resistance. The middle segment of NSY-Chr 14, where a locus to reduce insulin resistance was mapped, overlaps with the region where *Adp1n*, which decreases adiposity, was mapped, and may well be the same locus. The NSY allele at this locus decreases adiposity and improves insulin sensitivity, as shown by the lower body weight, smaller fat mass and better insulin sensitivity observed in R1 mice than in R2 mice. The proximal and distal segments of NSY-Chr 14, where loci for insulin resistance were mapped, overlap with the segments where *Nidd2.1n* and *Nidd2.2n*, respectively, were mapped. R0 possesses an insulin-resistance allele at two loci, *Nidd2.1n* and *Nidd2.2n*, and an insulin-sensitivity allele at *Adp1n* in the middle segment of Chr 14, while R1 mice possess an insulin-resistance allele at *Nidd2.1n* and an insulin-sensitivity allele at *Adp1n*, leading to less severe insulin resistance than that in R0 (Figure 4). Thus, the original *Nidd2n*, which was mapped to a relatively broad region in the middle portion of Chr 14 [6], is now dissected into multiple components: *Nidd2.1n* in the most proximal segment of NSY-Chr 14, retained in R2 and contributing to hyperglycemia, insulin resistance and adiposity; *Nidd2.2n* in the distal segment of NSY-Chr 14, retained in R0 but not in R1, for insulin resistance and hyperglycemia; and *Adp1n* in the middle segment of NSY-Chr 14, retained in R1 but not in R2, reducing adiposity and improving insulin sensitivity (Figure 4). The NSY alleles at *Nidd2.1n* and *Nidd2.2n* worsen metabolic phenotypes, while the NSY allele at *Adp1n* improves metabolic phenotypes by decreasing fat mass and improving insulin sensitivity.

Adipocytokines are important mediators linking adiposity with insulin resistance and metabolic abnormalities. Common forms of obesity are characterized by elevated circulating leptin [15]. Neither a high endogenous leptin level nor treatment with exogenous leptin is effective in ameliorating the most common form of obesity, consistent with a state of leptin resistance [16]. NSY mice had an extremely high level of leptin (Figure 3A), as was reported in a number of obesity models [17]. Leptin level in R0, R1 and R2 mice was significantly higher than that in control C3H mice (Figure 3A), indicating that NSY-Chr 14 harbors a locus for leptin resistance. Leptin level in R2 mice was as high as that in parental NSY mice, with no significant difference between them, while that in R0 and R1 was significantly lower than that in NSY mice. These data suggest that a locus contributing to leptin resistance is localized in the proximal segment of NSY-Chr 14 retained in R2. This region overlaps with the region for *Nidd2.1n*, affecting adiposity, insulin resistance and hyperglycemia, and may well be the same locus. The NSY allele at this locus appears to increase body weight and fat mass, and cause leptin resistance and insulin resistance, leading to hyperglycemia.

Since the serum level of leptin is known to be affected by fat mass, we studied the correlation of serum leptin level with fat-pad weight (Figure 3C). A significant correlation was detected in mice with C3H background (R0, R1, R2 and C3H mice) (Figure 3C, open circles). A significant correlation was also observed in NSY mice (Figure 3C, closed circles), but the regression line was markedly shifted toward the right. These results suggest that NSY-Chr 14 affected serum leptin level by increasing fat-pad weight, and that the NSY genetic background outside Chr 14 shifted the correlation between leptin level and fat-pad weight toward a lower level relative to fat-pad weight.

Studies of humans generally suggest that circulating levels of most adipocytokines are elevated in individuals with obesity. One exception is adiponectin, whose level is reduced in obesity [18] and plays a protective role against insulin resistance in vivo [19],[20]. In the present study, R0, R1 and R2 mice showed a markedly lower level of adiponectin than that in C3H mice (Figure 3B), indicating that NSY-Chr 14 decreases serum adiponectin level, as observed in human obesity. The fact that serum adiponectin level in R0, R1 and R2 mice was significantly lower than that in C3H mice indicates that the proximal region of NSY-Chr 14 retained in R2 contributed to the decrease in serum adiponectin level. This locus may well be the same as *Nidd2.1n*, which is associated with increased fat mass, insulin resistance, leptin resistance and hyperglycemia. In contrast to the low adiponectin level observed in R0, R1 and R2 mice, NSY mice showed a markedly higher level of adiponectin than that in C3H mice (Figure 3B) despite a much larger fat mass than that in C3H, R0, R1 and R2 mice (Table 1). A significant negative correlation between fat-pad weight and adiponectin level was observed in R0, R1, R2 and control C3H mice (Figure 3D), as in the case of human obesity, suggesting that NSY-Chr 14 affected serum adiponectin level by increasing fat-pad weight. In NSY mice, however, this correlation was completely lost (Figure 3D), indicating that the NSY genetic background outside Chr 14 contributed to the increase in serum adiponectin level and dysregulation of adiponectin level relative to fat-pad weight. These data, together with the data on leptin discussed above, suggest that NSY-Chr 14 affects adipocytokine level through changes in fat-pad weight, as is observed in human obesity, while the genetic background of NSY mice outside Chr 14 causes abnormality of adipocytokine regulation in that leptin level is shifted toward a lower level relative to fat-pad weight and the correlation between adiponectin level and fat-pad weight is completely lost.

The proximal region of Chr 14, where *Nidd2.1n* was mapped in the present study, was previously shown to be linked to diabetes and/or obesity in several other independent crosses [21]-[27]. The data in the present study together with previous reports strongly suggest the importance of the proximal region of Chr 14 in conferring susceptibility to diabetes, insulin resistance and fat accumulation common to several strains of mice. In humans, a locus for waist-hip ratio has been mapped to the syntenic region by GWAS study [1], and Stabilin 1 (*Stab1*; 19.09 cM; which acts as a scavenger receptor for acetylated low density lipoprotein) [28] is a candidate gene in the region. Other candidate genes on Chr 14 are a protein kinase C, delta gene (*Prkcd*; 18.82 cM; which has an important role in insulin receptor signaling) [29]-[32], a pancreatic polypeptide receptor 1 gene (*Ppyr1*; 20.80 cM; a synonym of neuropeptide Y receptor 4) [33], and a docking protein 2 (*Dok2*; 36.71 cM; a member of the insulin receptor substrate-1 family of proteins) [21],[34]. Type 2 diabetes and obesity are complex, heterogeneous disorders with strong genetic components. Identification of responsible genes and demonstration of their effect on disease development remain a formidable challenge. To identify causative variants, further studies with sub-congenic strains, gene expression profiling in target organs, as well as gene-targeted approaches, such as knockout and knock-in mice, are necessary [35].

## Conclusions

The present study demonstrated that *Nidd2n*, a locus for hyperglycemia and insulin resistance mapped to Chr 14 in our previous study, consisted of multiple components (Figure 4). *Nidd2.1n* in the proximal segment of NSY-Chr 14 increased body weight and fat-pad weight, caused insulin resistance, and increased post-challenge glucose levels, but decreased fasting glucose level. *Nidd2.2n* in the distal segment of NSY-Chr 14 contributed to fasting as well as post-challenge hyperglycemia and insulin resistance. *Adp1n* in the middle segment of NSY-Chr 14 contributed to reduction in body weight and fat-pad mass and better insulin sensitivity. It should be noted that this conclusion was drawn from the presumption that all QTLs act in an additive fashion, and the possibility of non-additive interaction among the loci cannot be excluded.

The congenic strains established in this study provide powerful tools for investigating the pathological and physiological consequences of individual susceptibility genes for diabetes and obesity. Subsequent construction of new sub-congenic strains and sequencing analysis will lead to fine mapping and identification of causal variants of responsible genes for *Nidd2.1n*, *Nidd2.2n* and *Adp1n*. In addition, a congenic study with gene expression profiling in target organs [36], i.e. adipose cells, liver, and pancreatic islets) will help to detect causative genes in the NSY mouse. Such studies are now underway [37].

## Abbreviations

Chr: Chromosome

ipGTT: Intraperitoneal glucose tolerance test

ITT: Insulin tolerance test

NSY: Nagoya-Shibata-Yasuda

QTL: Quantitative trait locus

## Competing interests

The authors declare that they have no competing interests.

## Authors’ contributions

NB performed the experiments and wrote the manuscript. HU, SN, YH, MIB, MK and TF participated in the acquisition of data. HI contributed to designing the experiment, interpreted the data and edited the manuscript. All authors have read and approved the final manuscript.

## Additional file

## Supplementary Material

Additional file 1: Table S1. Polymorphic markers used in this study. Click here for file
