# Supplementary material for: Genetic dissection of susceptibility genes for diabetes and related phenotypes on mouse chromosome 14 by means of congenic strains
Source: BMC Genet. 2014 Aug 29;15:93. doi: 10.1186/s12863-014-0093-8 (PMC4152764; doi:10.1186/s12863-014-0093-8)
Supplement: Additional file 1: Table S1. — Polymorphic markers used in this study. [file s12863-014-0093-8-S1.doc]

**Additional file 1: Table S1** Polymorphic markers used in this study.

| Chromosome | Marker | (cM) | Chromosome | Marker | (cM) | Chromosome | Marker | (cM) | Chromosome | Marker | (cM) |
| --- | --- | --- | --- | --- | --- | --- | --- | --- | --- | --- | --- |
| **Chr 1** | *D1Mit173* | (20.14) | **Chr 6** | *D6Mit273* | (22.51) | **Chr 11** | *D11Mit74* | (3.52) | **Chr 14** | *D14Mit206* | (11.53) |
|  | *D1Mit19* | (38.01) |  | *D6Mit178* | (42.99) |  | *D11Mit76* | (9.41) |  | *D14Mit207* | (12.07) |
|  | *D1Mit305* | (44.95) |  | *D6Mit52* | (62.56) |  | *D11Mit229* | (15.63) |  | *D14Mit209* | (15.06) |
|  | *D1Mit14* | (67.71) |  | *D6Mit14* | (77.64) |  | *D11Mit231* | (21.92) |  | *D14Mit186* | (16.80) |
|  | *D1Mit461* | (89.95) |  |  |  |  | *D11Mit236* | (27.39) |  | *D14Mit59* | (24.47) |
|  |  |  | **Chr 7** | *D7Mit20* | syntenic |  | *D11Mit314* | (31.05) |  | *D14Mit5* | (31.49) |
| **Chr 2** | *D2Mit2* | (4.24) |  | *D7Mit62* | (48.36) |  | *D11Mit242* | (39.47) |  | *D14Mit235* | (33.34) |
|  | *D2Mit296* | (21.81) |  | *D7Mit238* | (63.78) |  | *D11Mit156* | (39.47) |  | *D14Mit125* | (44.17) |
|  | *D2Mit37* | (44.13) |  |  |  |  | *D11Mit320* | (43.21) |  | *D14Mit266* | (64.86) |
|  | *D2Mit304* | (62.49) | **Chr 8** | *D8Mit171* | (11.43) |  | *D11Mit195* | (51.34) |  |  |  |
|  | *D2Mit51* | syntenic |  | *D8Mit208* | (42.45) |  | *D11Mit286* | (54.95) | **Chr 15** | *D15Mit113* | (18.93) |
|  |  |  |  | *D8Mit167* | (63.84) |  | *D11Mit70* | (58.90) |  | *D15Mit123* | (29.31) |
| **Chr 3** | *D3Mit117* | (1.96) |  |  |  |  | *D11Mit54* | (59.82) |  | *D15Mit42* | (55.72) |
|  | *D3Mit98* | (37.83) | **Chr 9** | *D9Mit229* | (26.83) |  | *D11Mit145* | (61.07) |  |  |  |
|  | *D3Mit257* | (66.69) |  | *D9Mit269* | (47.15) |  | *D11Mit301* | (75.93) | **Chr 16** | *D16Mit88* | (9.23) |
|  |  |  |  | *D9Mit311* | (68.74) |  | *D11Mit168* | (78.74) |  | *D16Mit4* | (25.43) |
| **Chr 4** | *D4Mit111* | (28.7) |  |  |  |  |  |  |  | *D16Mit158* | (45.89) |
|  | *D4Mit219* | (47.21) | **Chr 10** | *D10Mit194* | (24.48) | **Chr 12** | *D12Mit270* | (13.69) |  |  |  |
|  | *D4Mit48* | (73.41) |  | *D10Mit230* | (45.28) |  | *D12Mit255* | (37.82) | **Chr 17** | *D17Mit36* | (22.90) |
|  |  |  |  | *D10Mit164* | (67.32) |  | *D12Mit20* | (62.10) |  | *D17Mit206* | (45.20) |
| **Chr 5** | *D5Mit148* | (17.33) |  |  |  |  |  |  |  |  |  |
|  | *D5Mit41* | (50.68) |  |  |  | **Chr 13** | *D13Mit13* | (30.06) | **Chr 18** | *D18Mit35* | (23.86) |
|  | *D5Mit262* | (70.66) |  |  |  |  | *D13Mit74* | (56.92) |  | *D18Mit7* | (51.92) |
|  |  |  |  |  |  |  | *D13Mit78* | (67.21) |  |  |  |
|  |  |  |  |  |  |  |  |  | **Chr 19** | *D19Mit80* | (18.24) |
|  |  |  |  |  |  |  |  |  |  | *D19Mit34* | (50.72) |
|  |  |  |  |  |  |  |  |  |  |  |  |

The map positions of SSLPs (in parentheses) were obtained from the Mouse Genome Database updated on 04/29/2014 (http://www.informatics.jax.org).
